# Newly educated care managers’ experiences of providing care for persons with stress-related mental disorders in the clinical primary care context

**DOI:** 10.1371/journal.pone.0224929

**Published:** 2019-11-12

**Authors:** Lilian Wiegner, Dominique Hange, Irene Svenningsson, Cecilia Björkelund, Eva-Lisa Petersson

**Affiliations:** 1 Primary Health Care, School of Public Health and Community Medicine, Institute of Medicine,Sahlgrenska Academy, University of Gothenburg, Gothenburg, Sweden; 2 Institute of Stress Medicine, Region Västra Götaland, Sweden; 3 Research and Development, Primary Health Care, Region Västra Götaland, Sweden; Aga Khan University, KENYA

## Abstract

**Objective:**

Our aim was to explore how the care managers put the complex care manager task into practice and how they perceived their task, which was to facilitate effective, person-centred treatment for stress-related disorder concordant with evidence-based guidelines in primary care.

**Design:**

This was a qualitative study using examination reports from the course for care managers. Systematic text condensation according to Malterud was used for the analysis.

**Setting:**

Primary health care centres

**Subject:**

Twenty-eight newly educated care managers in primary health care participated in the study. The median age was 50 (31–68) years. Twenty-seven were women and one was a man. Twenty-one were employed as nurses and seven as counsellors.

**Results:**

The informants perceived the role as care manager as meaningful but at times complicated. To participate in teams and to work closely with the general practitioner was experienced as important. The co-ordinating function was emphasised as especially important, as well as the increased continuity in care. The dual role as care manager and counsellor was sometimes experienced as problematic.

**Conclusion:**

The informants took advantage of the knowledge they had attained during the course. They perceived themselves as being a bridge between patients and other professionals. The result of having dual roles at the primary health care centre unexpectedly revealed difficulties for some professionals. The nurses seemed more familiar with the new way of working.

## Introduction

Primary care is the major care sector for people with symptoms of depression, anxiety and stress-related mental problems, i.e. common mental disorders (CMD). In Sweden, around 70% of all patients with CMD problems are taken care of solely in primary care, representing around 10% of all visits per year to the primary care centre (PCC), yielding a comprehensive care responsibility for the personnel [1]. In working life today, CMD is one of the most common reasons for sick leave and is costly not only for the individual but also for society [2]. Onsite collaborative care organisational interventions at the PCC with the addition of a care manager, usually a part-time specialised nurse, is a cost-effective intervention that has been shown to improve the quality of CMD patients’ care [3–7].

Care management combines increased accessibility to the PCC with continuity of care for the patient and organisational and educational development at the PCC. The care manager puts collaborative care into practice by combining the responsibility for providing support and continuity to the individual patient with the provision of feedback on the course of the patient’s depression to the physician and communication with the care team. A care manager’s task/assignment is a complex intervention, requiring broad knowledge including knowledge about care of people with mental health problems, communication skills, and organisational skills. The cornerstone of care is the provision of an initial person-centred interaction with the patient where the patient’s ideas, concerns and expectations are elicited and developed into an individual care plan through mutual discussion and where the patient's needs are combined with the best accessible resources [8]. To provide continuity, follow-up can preferably be provided by telephone contacts, where the course of the patient’s recovery can be followed, and, especially in case of poor development, be communicated to other PCC personnel engaged in the patient’s treatment, e.g. physicians, psychotherapists, physiotherapists, and counsellors, depending on the local PCC and its resources [9]. In this way, the care manager not only provides care and continuity for the patient, but also constitutes prerequisites for an advanced diagnostic process during the rehabilitation period, as well as stepped care. Sweden has evidence-based national guidelines for the treatment of patients with depression and anxiety syndromes in primary care [10]. In 2015, the Västra Götaland Region started up an evidence-based implementation of a care manager for patients with depression, anxiety syndromes and stress-related mental disorder [3, 11]. Around 160 PCCs are now (2019) offering care manager contact to CMD patients. Completion of an academic course, Care Manager for CMD, 7.5 advanced education credits, is obligatory for care managers, prior to starting up the implementation of care management at the PCC. We set out to investigate whether the course gave prerequisites for conducting the complex care manager task by analysing written examination reports where the newly educated care managers described their care of a patient with stress-related CMD.

Our aim was to explore how the care managers put the complex care manager task into practice and how they perceived their task, which was to facilitate effective, person-centred treatment concordant with evidence-based guidelines in primary care.

## Methods

### Study design and participants

The inclusion criteria were the following: persons who had participated in a course for care managers for CMDs and who had written an examination report of their work as care manager for a patient suffering from stress-related mental disorder, and preferably exhaustion disorder. Our intention was to explore care managers’ experiences of how to provide care for such patients, after having completed the obligatory care manager course. The courses (Care Manager for CMDs in the Primary Care Context, with 7.5 advanced education credits) were arranged by the Institution of Medicine at the Sahlgrenska Academy of Gothenburg University, together with Primary health care. Applicants to these courses had to have at least a bachelor’s degree within health science or social work and to be registered nurses or counsellors with extensive experience of working with patients/care but new as care managers.

### Academic course for care manager for CMD

The course focused on the care of patients with mild to moderate depression, anxiety disorders and stress-related mental disorder in a primary care context where the care managers have an important role as giving support to the patients. The theoretical part in the course consisted of six days with seminars about CMD, rating scales and structured suicide assessment. One day was allocated to lectures on stress-related mental disorders and diseases, provided by the Institute of Stress medicine.

### Data collection

This is a qualitative study using written examination reports as the data source for the analyses. The examination report consisted of the care manager’s description of the care plan and reflections on the care process at the PCC. Thirty-six persons in the course for Care Manager for CMD were asked to participate in the study and 28 agreed to participate. Eight persons declined to participate for various reasons. Information was given to the informants about confidentiality and that participation would not affect their forthcoming work, and they signed a consent form.

Among the informants 27 were women and one was a man. Median age was 50 (31–68) years. 21 (75%) were nurses and seven (25%) were counsellors. In total, 28 informants were included in the study (see Table 1).

**Table 1:** Data concerning gender, age, working status for the 28 informants.

	Nurse	Counsellor	Total
	N (%)	N (%)	N (%)
**Age group**			
-25-45	8 (38)	1 (14)	9 (32)
-46-69	13 (62)	6 (86)	19 (68)
**Sex**			
-Women	20 (95)	7 (100)	27 (96)
-Men	1 (5)	0 (0)	1 (4)

### Analysis

Data were analysed by systematic text condensation (STC) according to Malterud [12], inspired by Georgi [13], i.e. developing descriptions and concepts concerning experiences of working as a care manager. STC was chosen because it aims to describe the informant’s experiences, as expressed by themselves, rather than to explore the possible underlying meaning of their statements. This method is often used in qualitative educational research and was found suitable for our research question.

STC involves four steps:

1. Reading all the texts several times to obtain an overall impression.
2. Identifying units of meaning, representing different aspects of the research question, and coding and sub-coding of these.
3. Condensing and summarising the contents of each of the coded groups.
4. Generalising descriptions and concepts reflecting the informants’ most important experiences as care managers.

The units of meaning in step 2 were systematically obtained by examining the text line by line, looking for content that could shed light on the objective of the study. The examination reports were coded, and the codes and subcategories were finalised after several discussions. Several meetings were held with all authors to discuss the results.

Two of the authors, a general practitioner (GP) and an occupational therapist, performed the analysis. Neither of them has clinical experience of working as care managers but both have lengthy experience of working in primary health care. The GP is also working as a senior physician at a specialist clinic for patients with stress-related mental disorders, such as exhaustion disorders.

### Ethical approval

The study was approved by the Regional Ethical Review Board in Gothenburg, Sweden, Etikprövningsmyndigheten (www.epn.se) (Dnr.903–13, Trial registration: Clinical Trials.gov Identifier: NCT02378272 February 2 2015.

## Result

The findings were summarised in terms of the following six code groups: the role of care manager, beneficial with collaborative teamwork, role conflict ?, individualised caretaking, the necessity of authority and consensus, and non-adherence to the methodology. Table 2 presents examples from the analysis.

**Table 2:** Examples of code groups and subgroups identified by text condensation.

Meaning units	Code groups	Subgroups
Through the care manager, the deteriorating mood could be quickly detected and the patient received the right help.	The role of care managing	Positive feeling being a care manager
It was difficult to help the patient fully when he/she had problems with focusing and finding time to improve his/her health.		Difficulties
It is also important to make an early assessment of the patient's potential for change, which I perceive is the most important prerequisite for successful treatment	Individualised caretaking	Alliance with patient
By showing regional medical guidelines regarding stress-related mental disorder and showing that there are other differential diagnoses that have not been investigated.		Take command over situation
In this case I had to use not only oral information but also to illustrate on a white board the different phases in stress-related mental disorder and to reassure the patient that she will recover but that it will take some time		Find new ways to reach out with their message
The self-assessment instruments give an initial insight into the patient's way of thinking and experiencing their situation		Use of self-assessment instruments

### The role of care manager

Participants experienced the care manager role as meaningful, but at times complex. The co-ordinating function was emphasised as especially important, as well as the possibility for an increased continuity in care. According to the participants, the same security and continuity could not have been achieved, if the patient had only seen a physician. It was especially important to be able to co-ordinate the resources, not least for patients with much anxiety.

“As care manager, I am the spider in the web, and I have close contact with the patient’s physician.” (Care manager (CM)11)

The participants perceived that they had the opportunity to detect deterioration at an early stage and thus to prevent more serious illness. Considerable time was spent on convincing the patient that a change was necessary. However, when taking everything into consideration, especially patients’ need for expressing themselves, participants experienced some lack of time.

“But it was difficult to keep to the 60 minutes allotted for conversation and documentation, especially when the patient needed so much time for the dialogue.” (CM26)

Telephone follow-ups provided flexibility, and many experienced this as particularly positive, as the patient group often showed cognitive difficulties and thus had problems remembering appointment times. The telephone follow-up was seen as less demanding than an appointment, especially when it was difficult to build a bond with the patient, e.g. when the patient did not accept the diagnosis or prognosis. The process surrounding the measures to be taken if the patient did not answer the telephone was experienced as a security.

### Beneficial with collaborative teamwork

Participants could at times experience the care manager role as insufficient; for example, they reported lack of time. Being surrounded by a well-functioning team, with a physician, psychologist, counsellor, rehabilitation co-ordinator, physiotherapist, and at times, an occupational therapist, helped to facilitate the work. The team was seen as together being able to provide a more complete caretaking, thus becoming a health promoting factor. Team members provided the care manager with an opportunity to get advice and to ease the burden. The team was at times organised as a psychosocial team and at times as a rehabilitation team.

“Getting support from the psychosocial team, which contained several different competencies, gave the patient a sense of security and she felt well taken care of, that we were there and could answer all questions.” (CM13)

Regardless of the organisation, it was necessary to have a close collaboration with the treating physician, especially when the care manager was new to the role. Further, working at a health care centre with many medical locums posed difficulties for the care manager, since reciprocal trust and a mutual action plan were required. In cases where the patient had difficulties letting go of the idea of physical illness and also when there was co-morbidity with other mental illness, it was extra valuable to have a good dialogue with the physician.

“It is crucial to have an understanding and competent physician to work together with–one who understands what recurrent depression with co-morbid fatigue syndrome is, and that rehabilitation takes a long time.” (CM28)

Co-operation with regard to sick leave listing was experienced as both necessary and valuable. The physicians made it known to the care managers that the information that they received about the patient through the new way of working made it easier for them to compose the medical grounds for sick leave listing.

### Role conflict?

Some participants had dual roles at the healthcare centre, since in parallel with the care manager job, they were working as counsellors, psychotherapists, Cognitive behavioural therapy (CBT)-therapists or rehabilitation co-ordinators. These participants felt that their roles overlapped, which meant that at times the care managers did their mapping according to learning psychology principles, or provided deeper conversational support, or gave exercises for managing anxiety, which was not the idea behind the care manager function. Some participants experienced the care manager role as completely incorrect and regarded the entire care manager function more as an alternative to the role of counsellor, rather than as basic and essential caretaking.

“I myself am not completely satisfied at this point because my experience is that in the long run, patients with long-term sick leave benefit from therapy on a regular basis, from learning how to manage emotions (cognitive training) and understanding the reasons why they are ill.” (CM20)

Some of the participants who were both care managers and therapists were unwilling to change the form of the contact from a more therapeutic contact to a contact with focus on care co-ordination. Clarity towards the patient was deemed necessary, and participants expressed the desire for getting help during the training with unifying the two roles.

“What gets difficult in this dialogue is that I cannot relinquish my role as psychotherapist, so it turns into a mixture of the care manager function and the role as psychotherapist.” (CM24)

There were participants who experienced that they had to collaborate with themselves, and thus they had less time for their therapeutic “non-care manager tasks”, which they were not satisfied with.

“Since I also work as a CBT-therapist, a part of the internal collaboration is with myself, so to speak. There are advantages with this, but unfortunately this opens up for the possibility that the group that we wanted to have increased time for, ends up having less time, and this was not the intention with the implementation of the care manager function.” (CM31)

### Individualised caretaking

The knowledge transfer between the care manager and patient was based on the notion of providing help to self-help. In order to achieve this, it was necessary to be sensitive to the patients’ needs and to have an individualised care orientation.

“The focus is on the patients’ need of help and together we strive towards reaching interim goals and main goals.” (CM11)

Participants worked basically according to the template for the care manager task. After mapping out the patient’s needs, the participants informed them about possible primary health care efforts, with the aim of encouraging lifestyle changes. In addition, structuring up stress factors gave patients the possibility to gain insight into their entire life situation, and they were given a manifest participatory role in the rehabilitation.

“She needed someone to help structure up all of the stress factors and to filter away those that were unnecessary to put energy into. This became a change for the patient that enabled her to be able to work further and to focus on what was important.” (CM23)

The assessment form was very helpful for the participants when they had to take stock over the patient’s need of help. The instrument was experienced as positive, even though initially it felt unfamiliar to work according to a template. After having made an inventory of the patient’s motivation for change, it was important to find a good balance between rest and activity in order to succeed with the rehabilitation.

Sometimes it was necessary to provide the same information repeatedly because patients could not readily absorb the message, often due to cognitive difficulties. An alliance with the patient was the very foundation of the contact, but at times the participants experienced that they had to take command in order to make it easier for the patient.

“Basically, it is meaningless to even believe that recovery can take place if she cannot be completely free from formalities for a while, since these are obviously causing her a great deal of stress. Therefore, it is necessary to take command in order to improve the patient’s understanding of the situation” (CM31)

Participants made use of medical guidelines to persuade the patient about the importance of treatment. In certain cases, both oral and written information was used, but also illustrations on a white board. Participants also described that the new way of working was an opportunity to increase the competency of their co-workers.

### The necessity of authority and consensus

Caretaking was influenced by the care manager’s own view of the patient’s need and the degree of suffering. An authoritative approach, rather than consulting with the individual, was seen as appropriate mainly when it was difficult for the patient to accept being put on sick leave.

“At the first visit to the doctor the patient did not want to be on sick leave, even though the doctor suggested that. After a conversation with the care manager the patient agreed that she needs to be home from work for some time.” (CM21)

The same approach was applicable when the participant had difficulty believing in the patient’s symptoms. This was particularly clear in a case where the participant felt that a patient with substance abuse was using her to get drug prescriptions.

A combination of authority and consensus was often used in the contact, and a certain amount of persuasion could become necessary before the patient accepted the treatment ordinated by the physician and follow-up.

### Non-adherence to the methodology

In certain cases, the participants actively chose not to follow the methodology. The reason for this varied. When anxiety was not present in stress disorder, for example, the GAD-7 was not used. At times the participants perceived that they had sufficient information without using KEDS, while at other times there was the feeling that the patient would not answer the questions truthfully.

”KEDS-9 was not used as I perceived that the other assessment forms gave me enough information to support help to self-help.”(CM7)

When the patient was critical to the diagnosis, the participants chose in certain cases to delay the information about rehabilitation efforts such as stress management courses, as this did not seem meaningful.

## Discussion

### Main findings

Twenty-eight newly educated care managers in PCCs described in their examination reports their experiences of providing person-centred care for patients suffering from stress-related mental disorders. This qualitative study explored their perceptions about care provision, as well as perceived benefits and challenges in their new role of supporting patients suffering from high stress load. According to the results, they perceived the role of care managing as meaningful, but at times complex. The co-ordinating function was emphasised as being especially important, as well as the increased continuity in care. Some of the informants worked in teams but all found it important to work close to the GP. The participants wanted to support help to self-help and found their role highly important regarding patients on sick leave as well as distressed patients. To use questionnaires and telephone follow-ups was perceived mostly positive, but it seemed important with flexibility. Thus, it was also important to have the possibility to go outside the structures at times, if the patients had other needs. Care managers with dual roles, also working as a counsellor at the same workplace, sometimes found their role as care manager as less suitable for the patient. Lack of time was an important negative aspect even to nurses.

### Comparisons with previous research

The role of care manager was complex and involved providing supportive person-centred care as well as acting as the bridge between patients and other professionals. It is well known that person-centred care engages the patient as an active partner [14]. Person-centred care addresses a person's belief in the ability to successfully execute the behaviours necessary to produce desired outcomes, instead of attempting to force patients towards certain activities. [15]. Caregivers perceive less stress when working after the person-centred care concept [16], and patients perceive less anxiety and depression. The task of providing person-centred care was not new for the participants, who had been working in primary care for many years. A recently published study has shown that having someone to share with could alleviate the patient’s burden [17]. During their education the care managers were informed that a trusting relationship between the care manager and the patient was important. Despite this, the role as a trusting partner to the patient was not emphasised in the written examination reports. This aspect might have been obvious to the care manager, and therefore they failed to mention it. However, the main focus for the care managers seemed to be to help the patient to identify their stress load, support them in their struggle to make the necessary changes in their lives and to offer help from other professionals, if necessary.

To map the patient’s situation and as a complement for further discussions, the care manager was educated to use self-assessment instruments regarding stress, depression and anxiety. The use of assessment instruments in PCC is not obvious [18], but Petersson et al [19] found that GPs perceived them useful as a starting point for discussion as well as to facilitate communication with actors outside PCC such as insurance offices. In this study the main purpose of the self-assessment instruments used by the care manager was to facilitate discussion on improvement in health. Most of the care managers perceived the use of instruments as positive; it gave a sense of stability, even though working according to a template sometimes felt unfamiliar. The participants perceived that the patient felt involved in their treatment, and the instruments were a way of mapping the patient’s difficulties and a help for self-reflection. Sometimes care managers chose to ignore the assessment forms. One reason for this was that they perceived that they had sufficient information without the form. In these cases, it was obvious that the care manager did not understand the advantage for the patient of using the form as a basis in the follow-ups.

Using self-assessment instruments as well as regular telephone follow-ups were new to most of the care managers. Follow-ups by telephone are previously described regarding chronic diseases and depression, in a context as part of a telehealth intervention, for example, concerning internet CBT [20]. Salisbury et al [21] showed improvements in self-management and reduced anxiety and depression by using regular telephone calls to patients suffering from depression. This is of importance, since anxiety syndromes as well as depression are common among patients suffering from stress-related mental disorders [22]. Telephone-based collaborative care strategy for delivering care for anxiety disorders has been shown to broaden mental health-related quality of life [23], which was why it was important to use telephone follow-ups as a part of the mapping. The care managers in this study perceived that telephone follow-ups were less demanding and less time consuming than appointments. This way of working gave a possibility of not letting go of patients with difficulties in accepting their diagnosis and those with huge cognitive impairments.

The care managers used different approaches to reach the patients. In cases when the patient did not accept being put on sick-leave, they found it important to be authoritative, but otherwise they consulted with the individual as advocated in person-centred care. To offer best possible caretaking, some of the care managers perceived it important to have an organisation with teams containing psychologists, rehab coordinators, as well as physiotherapists. Previous studies confirm that working as a team sometimes could be successful in primary care [24], provided that the organisation of clinical practice is functioning [25]. In our study the team was perceived as advisory to the care manager, as a way to ease the burden, as well as a possible complement for the patient’s treatment. For example, the patients did not have to repeat their story from the beginning to new professionals. The care manager found it important to be a link for patients who were tired and not in a position to struggle for their needs.

Further, it was important that the care managers identified the task as care manager as that of providing supportive care but not therapeutic interventions, as some care managers indicated problems with separating the role as care manager and therapist. The dual roles could become a problem. Counsellors mapped according to psychological principles, to give advice and to provide deeper conversational support. However, the care manager’s role is to act as a support, rather than to be a caretaker of the patient’s disorder. Therefore, it is important that the care manager is clear about the need for a realistic level of commitment in the patient’s problem, which was something some of the care managers struggled with.

Most sick-leave listings in Sweden are dispensed by GPs within primary health care. To provide requested and accurate information in the sickness certificate is a challenging task for physicians [26]. The care managers perceived that they were able to provide detailed information about the patient’s functional impairment, which facilitated the physicians’ certificate writing, as well as made the patient less anxious. To cooperate with the responsible physician was of highest importance to the care managers, but also to the patients. Udo et al [17] found that the patients perceived that the care manager’s close contact with the physician was most important.

Working at a PCC with many medical locums was a problem since a close collaboration with the treating physician was perceived as essential. On the other hand, under these circumstances, the presence of a care manager was essential for the patient. The care manager could partly compensate for poor contact with the physician and in that way provide a better continuity of care.

In addition to lack of continuity regarding doctors, several of the care managers perceived lack of time as a problem. This problem could arise from difficulties within the organisation but might also be due to the need to accumulate sufficient time for the method to become settled. We believe that it is necessary to have an organisational leader who supports changes and sets aside time for the assignment. To what extent this was the case among the participants remains uncertain.

Another factor contributing to time constraints was the extra time needed for patients suffering from cognitive impairment. This is a common problem regarding stress-related mental disorders, such as exhaustion disorder [27]. Due to these impairments the information had to be repeated several times, which of course was time consuming. This highlights another important advantage with the care management organisation, namely the continuous contact with the patient and possibility to repeat information. Nevertheless, time is unfortunately scarce in primary care, and this of course could be a problem when implementing care management at PCCs.

### Strengths and limitations

This study was performed among nurses and counsellors employed in primary health care, which represents the “real world” clinical context in Sweden. The sample was large enough to represent different views from the participants in the course. This indicates that our findings have validity. The data was informative and adequate, providing coherent stories containing abundant and diverse accounts of what we intended to explore, which is more important than getting saturation [12]. It is likely that the participants presented truthful reports, since they described real encounters from practice with patients. Therefore, the trustworthiness was established. The findings might not to be fully transferable, as for the student the aim was to be a care manager and for the researcher the aim was to gain knowledge of the students’ learning and its implication.

The authors have lengthy experience working in primary health care and might have preconceived notions. To avoid bias, reflexivity was used continuously throughout the process by discussing the procedure with the research team [12] The examination reports used for the analysis might differ from the care manager’s ordinary way of working, which could be a limitation. However, this was not possible to confirm. Only 4 percent of the informants were men, and this is fewer than the gender distribution of nurses in Sweden, since 12% of the nurses in 2017 were males.

### Implications for practice

This study shows that the care managers provide care in line with the education given in a care manager course in person-centred care. The use of a care manager could therefore be a good supplement in the treatment of these groups of patients. However, more studies are needed to validate these results.

## Conclusions

This study shows that the informants took good advantage of the knowledge they attained in the course. After the course they perceived themselves as being the bridge between the patient and other professionals, providing comfort to the patient.

Having dual roles at the PCC unexpectedly revealed difficulties for some professionals, but the nurses seemed more comfortable with the new way of working.

These findings are valuable and should be considered in future educational efforts.
